# Effects and Characterization of Some Topical Ointments Based on Vegetal Extracts on Incision, Excision, and Thermal Wound Models

**DOI:** 10.3390/molecules25225356

**Published:** 2020-11-16

**Authors:** Calin Vasile Andritoiu, Corina Elena Andriescu, Constanta Ibanescu, Cristina Lungu, Bianca Ivanescu, Laurian Vlase, Cornel Havarneanu, Marcel Popa

**Affiliations:** 1 Apitherapy Medical Center, Balaneşti, 217036 Gorj, Romania; dr_calin_andritoiu@yahoo.com; 2 Nutrition and Dietetics Specialization, Faculty of Pharmacy, Vasile Goldis Western University of Arad, 86 Liviu Rebreanu Street, 310025 Arad, Romania; 3 Department of Morphofunctional Sciences I—Histology, Grigore T. Popa University of Medicine and Pharmacy, 16 Universitatii Street, 700115 Iasi, Romania; andriescu_corina@yahoo.co.uk; 4 Sf. Spiridon Emergency County Hospital, Department of Pathology, 700111 Iaşi, Romania; 5 Department of Natural and Synthetic Polymers, Faculty of Chemical Engineering and Environmental Protection, Gheorghe Asachi Technical University of Iasi, Prof. D. Mangeron Blvd., 700050 Iasi, Romania; cibanescu@tuiasi.ro (C.I.); marpopa2001@yahoo.fr (M.P.); 6 Department of Pharmaceutical Botany, Faculty of Pharmacy, Grigore T. Popa University of Medicine and Pharmacy, 16 Universitatii Street, 700115 Iasi, Romania; 7 Department of Pharmaceutical Technology and Biopharmacy, Faculty of Pharmacy, Iuliu Hatieganu University of Medicine and Pharmacy, 8 Victor Babes Street, 40010 Cluj-Napoca, Romania; laurian.vlase@yahoo.com; 8 Faculty of Psychology and Education Sciences, Alexandru Ioan Cuza University, 700554 Iasi, Romania; hcornel@uaic.ro; 9 Academy of Romanian Scientists, Splaiul Independentei Street, No 54, 050094 Bucharest, Romania

**Keywords:** wound healing, *Achillea millefolium* L., *Calendula officinalis* L., *Arctium lappa* L., *Hippophae rhamnoides* L., HPLC analysis, rheological characterization

## Abstract

The present paper aims to formulate and characterize four phytotherapeutic ointments based on *Hippophae fructus*, *Calendulae flos*, *Bardanae folium*, and *Millefolii herba*, which are included in a novel ointment base. In order to investigate the healing properties of the ointments, in vivo experimental wound models of linear incision, circular excision, and thermal burn were performed on Wistar rats. Topical treatment was performed daily for 21 days. Determination of the wound contraction rate (WCR), the period of reepithelization, and histopathological examination were achieved. Additionally, for the tested ointments, oscillatory and rotational rheological tests were carried out, and for the extracts, HPLC analysis was performed. The results demonstrate that the tested novel ointments are safe for use and the most effective ointment proved to be the one based on *Arctium lappa*, followed by that of *Calendulae flos*.

## 1. Introduction

Nowadays, the use of various complementary therapies has become increasingly popular and a frequent method of treatment. In this context, the plants used for the treatment of various diseases play a significant role and treating skin lesions with natural products is a promising research area. Although in recent years mortality rates caused by burns have decreased, there are still concerns related to wound infection, the formation of scarring, or to skin graft rejection [1]. Since treating injuries involves specialized medical service and high costs, it is beneficial to find new treatment methods based on natural products. Considering the high costs of synthetic and biosynthetic occlusive dressings [2], natural products could provide useful alternatives for the local treatment of cutaneous lesions in a less traumatic way.

Skin care products used in case of burns, trauma, and wounds are aimed to heal the skin quickly and to restore it without leaving keloid scars. Some of the pharmaceutical substances for external use, administered locally to treat skin lesions, can give cutaneous adverse reactions (irritation, itching, burning, rashes). It was observed that sometimes, long-term topical application for a long time of preparations based on silver sulfadiazine could cause side effects and even systemic effects (leucopenia, thrombocytopenia) [3].

Regarding the use of plants in the treatment of skin lesions, the therapeutic effects of sea buckthorn extracts (*Hippophae rhamnoides* L.) and marigold (*Calendula officinalis* L.) are highlighted. The oil obtained from the fruit and seeds of sea buckthorn is used to treat eczema, wounds that are difficult to heal, inflammatory skin diseases, burns, and thermal injury by low temperatures [4], and skin tissue irradiation. The literature recommends *Calendula officinalis* flowers in dermal and mucosal inflammation, furunculosis, bruises, contusions, burns, and wounds. This plant presents antiseptic, antioxidant, and anti-inflammatory properties, and it is often used externally to treat skin conditions [5]. Less studied in this regard are *Arctium lappa* L. (burdock) and *Achillea millefolium* L. (yarrow), which have anti-inflammatory effects [6,7]. Traditional medicine recommends burdock leaves for the healing of wounds, burns, and gastric ulcers [8].

From this perspective, scientific interest in finding a new formula based on plant extracts with healing, antimicrobial, and anti-inflammatory effects, which helps regenerate damaged skin, is in accordance with today’s research. In this context, the aim of this study is the formulation, characterization, and evaluation of four phytotherapeutic ointments to treat three types of skin lesions (incision, excision, and thermal burn). 

## 2. Results

### 2.1. Determination of Total Polyphenol Content

**Figure 1:**
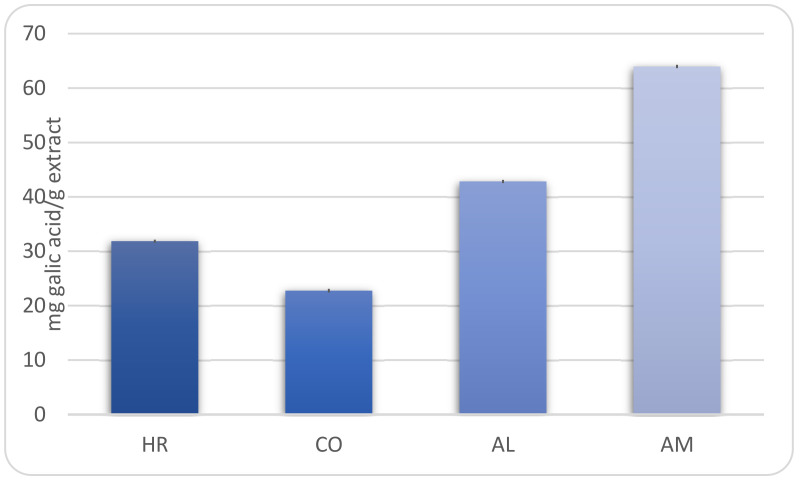
The total phenolic contents in hydro-alcoholic extracts (HR—*Hippophae rhamnoides*, CO—*Calendula officinalis*, AL—*Arctium lappa*, AM—*Achillea millefolium*).

Our study has revealed that the level of polyphenols ranged between 22.76 ± 0.33 mg gallic acid/g for *Calendula officinalis* extract and 63.99 ± 0.31 mg gallic acid/g for *Achillea millefolium* extract (Figure 1). 

### 2.2. HPLC Analysis

The phenolic profile of the extracts determined by HPLC is listed in Table 1. The results indicated the presence of caffeic acid, chlorogenic acid, and rutoside in all extracts. The analysis of methoxylated flavonoids allowed their identification only in *Achillea millefolium* extract, as follows: acacetin (107.12 ng/mL), casticin (63.56 ng/mL), and hispidulin (877.78 ng/mL) (Table 2). Sterol analysis showed that the dominant sterol in the three vegetal extracts is beta-sitosterol (Table 3). In *Achillea millefolium* extract, stigmasterol, campesterol, and brassicasterol were also identified, while in *Calendula officinalis* extract, stigmasterol and campesterol were quantified.

### 2.3. Rheological Characterization

Prior to all the other oscillatory tests, an amplitude (strain) sweep was performed to establish the limits of the linear viscoelastic range (LVR). Moreover, additional information about the structural stability for all samples could be obtained. The rheological tests were performed at two different temperatures: 25 °C (room temperature) and 37 °C (physiological temperature). In this test, the frequency was kept constant (ω = 10 rad/s), and the deformation was varied between 0.001% and 100%.

**Figure 2:**
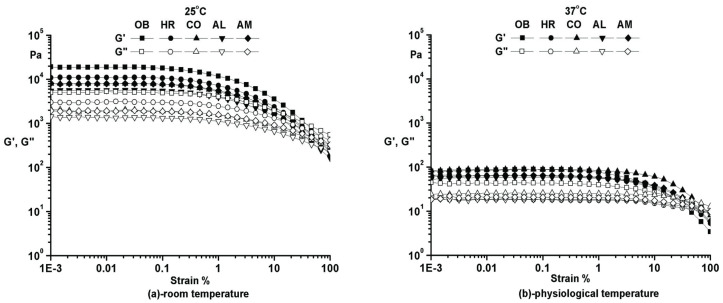
Amplitude sweep for the phytotherapeutic products series.

As can easily be noticed from Figure 2a,b, at both temperatures, all samples exhibit remarkable structural stability in the small deformations range. The storage modulus higher than the loss modulus indicates a well-developed network and a solid-like behavior. At 25 °C, all samples have higher storage modulus (G′), are stiffer, sensitive to small variation in deformation, and somewhat brittle. At 37 °C, the ointments are softer, smoother, and easy to apply on skin with extended LVR and low values for G′. Plant additives have no significant influence on the rheological behavior of the analyzed compositions, only a minor reduction in the dynamic moduli being observed.

**Figure 3:**
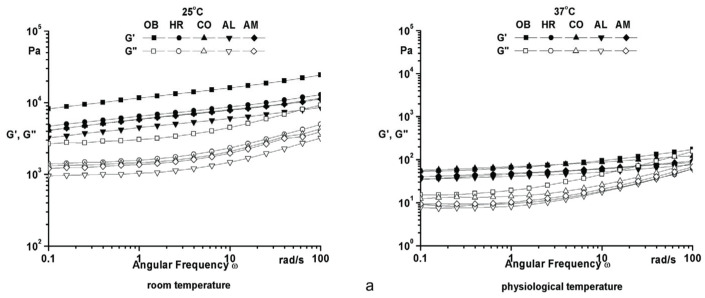

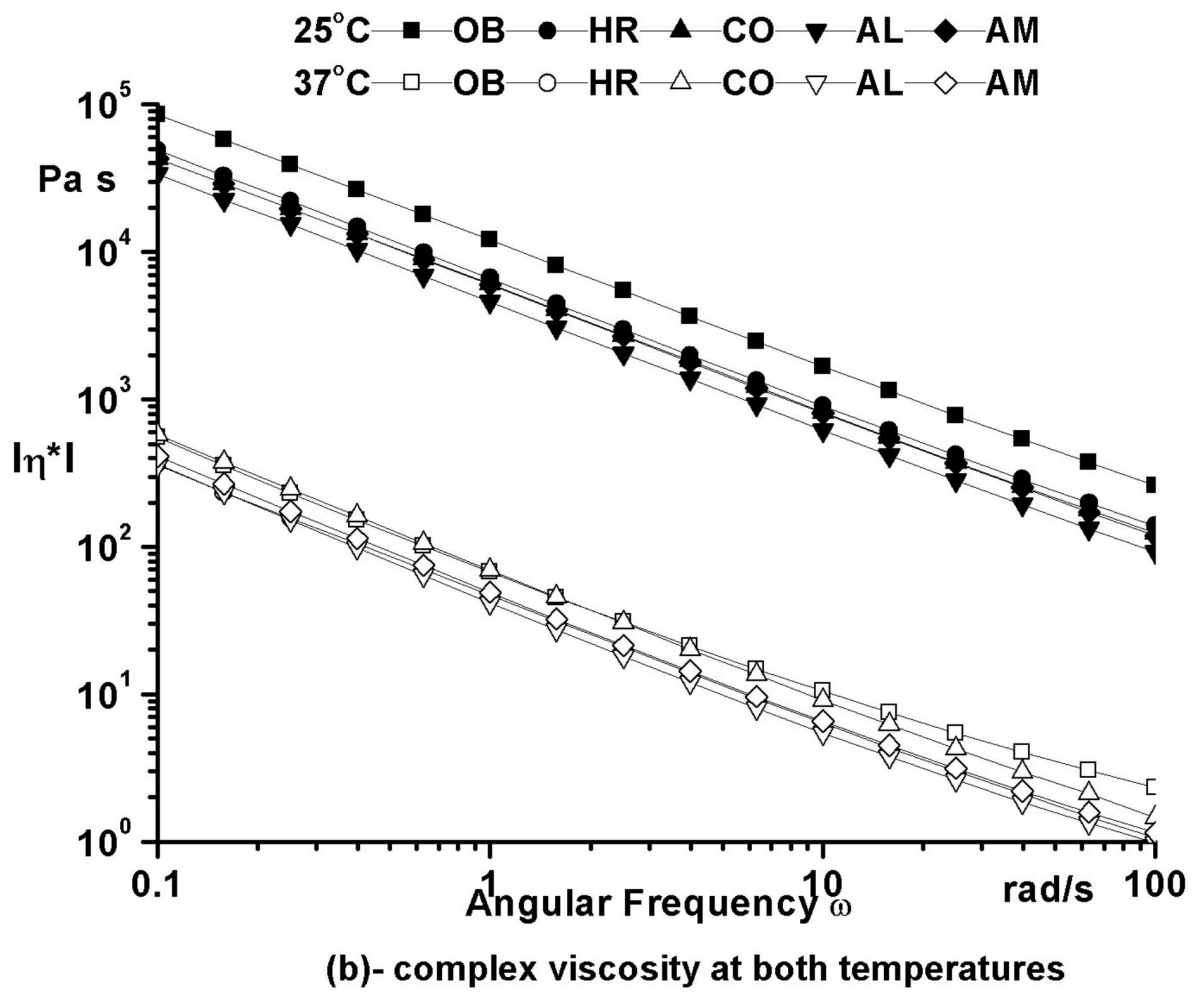
Frequency sweep of phytotherapeutic products series.

The frequency sweep test is widely used to obtain valuable data regarding the structural stability to evaluate the consistency at rest, long-term behavior, and separation behavior of materials. For the frequency sweep tests, the amplitude was kept constant within the linear viscoelastic range (γ = 0.05% at 25 °C, and γ = 0.1% at 37 °C), and the frequency was varied between 0.01 and 100 rad/s. As estimated, a solid-like behavior (G′ higher than G″—loss modulus for the entire experimental frequency domain) is characteristic for both temperatures, so all samples have stable structures without separation of components (Figure 3a). Decreased values with almost three orders of magnitude both for G′ and G″ at 37 °C suggest that the ointments are softer, smoother, and therefore easier to apply on the skin. High values of complex viscosity at low frequencies (Figure 3b) support the idea of stable structure at rest and a good spreadability on skin. No significant differences in the rheological behavior for the analyzed samples were noticed when plant additives were used.

**Figure 4:**
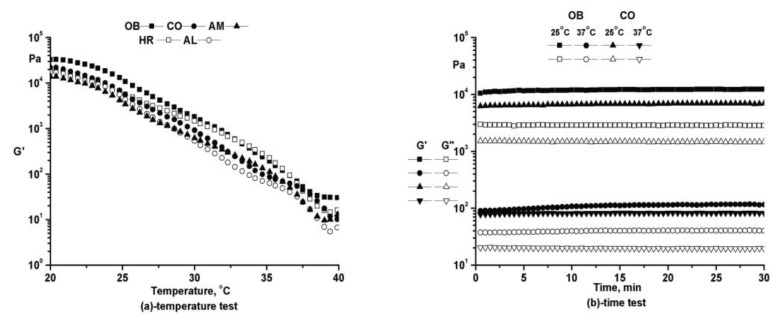
Oscillatory rheological tests of phytotherapeutic products series.

The temperature sweep test covering temperatures between 20 and 40 °C with a heating rate of 0.5 °C/min (performed at constant frequency ω = 10 rad/s and constant amplitude within the LVR as determined from the strain test) proved ointments fluidization around the physiological temperature, structure stability for all temperature range, and the spreadability of all formulations (Figure 4a).

The stability in time of the ointments structure was demonstrated by the time tests performed at both temperatures (25 and 37 °C), at constant frequency (ω = 10 rad/s) and constant amplitude within the linear viscoelastic range (Figure 4b). The results suggest the absence of separation or destructuration processes during storage.

**Figure 5:**
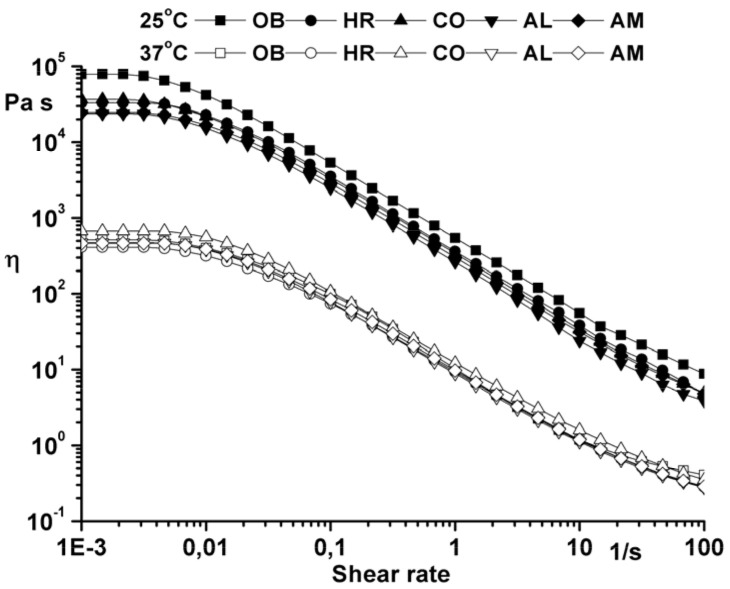
Flow curve of the analyzed ointments.

Flow curves (Figure 5) were recorded using a shear rate between 0.001 and 100 s^−1^. High zero shear viscosity values calculated with the Carreau–Yasuda model (η_0_ = 8.2·10^4^ Pa at 25 °C and η_0_ = 5.09·10^2^ Pa at 37 °C for the ointment base and the same order of magnitude for samples with plant additives) highlight the long-term structural stability of the studied ointments.

### 2.4. Wound Healing Evaluation Parameters

#### 2.4.1. Period of Re-Epithelialization and Wound Contraction Rate (WCR)

The re-epithelial period for excision was observed on the 12th day of treatment for all ointments tested as compared to the ointment base (OB) group (day 18 of the experiment) or to the negative control (NC) group (day 21 for normal healing). The re-epithelial area for the ointments is different (Table 4), the best result being recorded for the *Arctium lappa* ointment (1 × 2 mm).

The wound areas were measured at days 0, 6, and 9. The results are listed in Table 4 and are expressed as mean ± standard error of mean (SEM). No significant changes were recorded regarding the contraction of the wound during the first three days of treatment. During this period, a series of perilesional inflammatory processes and edema suggestive for the thermal burn injury could be identified. The proliferation of cells began after 3 days, and significant wound area reduction (*p* < 0.001) was achieved during day 6 and day 9. The AL group showed a wound contracture of 94.29 ± 0.26, which is comparable to that of the CO group (92.70 ± 0.47). Equally promising results were also obtained for the HR group (88.23 ± 0.49) and AM group (87.50 ± 0.41).

#### 2.4.2. Histological Examination 

The micrographies presenting the histopathological results after 3, 6, 12, and 21 days are given in Table 5, Table 6, Table 7 and Table 8.

The groups treated with herbal ointments for the incision type lesion (day 3) show a moderate chronic inflammation of the dermis and hypodermis, periadnexal (AL group), perivascular and congestive vessels (AM group). Inflammatory elements were present in the muscular plan (CO group, AL group, AM group).

In addition, in the excision type lesion, diffuse inflammatory elements around the vessels (CO group) were found in dermis and hypodermis, and ulceration starting from the epidermis to the deep (AM group) dermis. Fibrosis of the hypoderm all the way to the deep dermis and the presence of lymphocytes in the muscular layer were present in the case of the thermal burning lesions. For control groups (OB group and NC group), the situation of this day is similar, but with a much sharper picture, especially for the untreated group. In this case, abscess and ulceration of the epidermis were observed in the incision lesion, purulent exudate was observed in the excision model, and bacterial colonies accompanied by ulceration were noted for the burn injury.

Morphological findings approaching the normal were identified on day 6 for the AL group. Thus, the inflammatory infiltration of the muscular plane (incision model) was gone, granulation tissue underwent connective tissue organization (excision model), and for the thermal burn model, the annexes showed no modification.

Slight dermal collagenization in the incision lesion, an area undergoing connective organization in the excision lesion and the presence of inflammatory elements in the thermal burn model were the findings for the HR group. 

In the treated groups (HR group and AM group), the morphological aspects suggested a favorable evolution toward the improvement of the inflammation and repairing of tissues, but with the presence of inflammatory elements.

Vacuolar degeneration in the incision lesion, spongiosis, edema, and inflammatory elements in the excision lesion and the ulceration of the epidermis in thermal burn model are noted for the NC group.

Epidermis ulceration, hemorrhage, and abscess in the excision lesion, respectively thermal burn lesion, and a keratin foreign-body giant cell in the incision lesion could be noticed for the OB group.

For the batches treated with plant-based ointments, the histological findings on day 12 consisted of dermal collagenization and maturing granulation tissue. In the HR group, inflammatory elements for the three experimental models are still present. The presence of lymphocytes in the superficial dermis was also observed in the AL group.

In the dermis and hypodermis of the burn injury control groups, an inflammatory lymphocytic infiltration could be found, all the way up to the muscular plane. In addition, acanthosis and vacuolar degeneration were present in the burn type injuries of these groups. For the incision and excision lesions, foreign-body giant cells are observed in the control groups.

The group treated with ointment based on sea buckthorn, in all three models—excision, incision, and thermal burn—reveals mature granulation tissue with little blood vessels, rare inflammatory elements (lymphocytes, fibroblasts), and fibrosis. The granulation tissue occupies the full dermal thickness in excision, 1/3 in incision, and 2/3 of the dermis in the thermal burn model to leave room for the rest of dense fibrosis.

After 21 days of treatment with the marigold ointment, the findings consisted in the presence of mature granulation tissue in the excision lesion (about 2/3 of the dermal thickness), the incision lesion (1/3), and in the thermal burn (1/3). In the incision lesion and thermal burn, good collagenization is present in 2/3 of the deep dermis thickness, and the epidermis is completely regenerated in all of the three experimental models.

Significant dermal collagenization with the reduction of cutaneous inclusions for the incision type lesion, and mature granulation tissue for the excision type lesion and thermal burn is noted for the batch treated with burdock-based ointment. 

For the batch treated with yarrow-based ointment, the cutaneous fragment of the incision lesion is normal. In the excision lesion, marked dermal collagenization could be observed, and fibrosis and collagen densification in 1/3 of the dermis was noted for the thermal burn.

For the batch treated with an ointment base, and also for the untreated batch, numerous lymphocytes were observed for all types of lesions. For the incision-type lesion treated with an ointment base, a foreign-body giant cell could be noted. Dermal collagenization with dermo-hypodermic lymphocytic inflammation is present in the excision-type lesion, respectively thermal burn. In the incision-type lesion vascular congestion, superficial dermal collagenization and the presence of lymphocytes as well as foreign-body giant cells were observed. In the excision lesion, the presence of lymphocytes and high dermal collagenization with a fibrotic appearance was found. This type of process could be noticed in the burn injury, where the fistula is replaced by fibrosis (up to the level of the hypodermis). In the burn model, there is evidence of edema. The presence of numerous lymphocytes up to the muscular layer is noted in the incision lesion for the NC group. In the excision lesion, lymphocytes and edema represent the major findings, and in thermal lesion, dermal collagenization, edema, and the presence of myocytes. All of these findings, and the results obtained after the comparison with the test creams, highlight the favorable effect of the ointments and all the processes that have been controlled or stopped in the skin tissue.

## 3. Discussion

Wound healing is a complex mechanism that involves a process of reepithelialization, formation of granulation tissue, remodeling of extracellular matrix [9], and wound contraction [10]. Fibroblasts, leukocytes, and monocytes participate in this process and help to restore damaged skin appearance [11].

Studies on sea buckthorn oil have shown its effectiveness in the treatment of wounds and atopic dermatitis [1,12]. The wound-healing activity is correlated with the active principles, namely vitamins E and C with an important role in wound healing, vitamin K (prevents bleeding), carotenoids (stimulate skin epithelialization of skin tissue and mucous membranes), phytosterols (with antimicrobial effects and anti-inflammatory properties), and palmitoleic acid (promotes the epithelialization of skin tissue and mucous membranes via modulation of microcirculation) [13,14]. 

Burning lesions treated with sea buckthorn oil experienced a rapid contraction possibly due to an increase in the proliferation and transformation of fibroblasts into myofibroblasts [15] and a faster reduction in lesion area compared with silver sulfadiazine ointment [16]. Our results correlate with literature data concerning a good wound contraction rate, efficient dermal collagenization, with normal granulation tissue undergoing maturation.

During the experiment, in the three models, we observed at the dermis and hypodermic levels the presence of chronic-type inflammatory elements, and histopathological events were recorded in the first three days after the induction of injuries. Moreover, the inflammation in the excision and thermal burn lesions can be observed down to the muscles. The group treated with marigold ointment presents moderate inflammatory infiltration with normal aspects of fragment muscle. This shows the anti-inflammatory effect of the topical ointment used and its action within the deeper layers of skin tissue, exceeding them, showing effects even at the muscle levels. Granulation tissue undergoing maturation starting from the epidermis to the muscular layer is found in excision. In our study, we observed a remission of the inflammatory event after the third day for all the experimental models used in this study, confirming the data presented in the literature [17]. Marigold flowers have been widely used as an anti-inflammatory agent for the treatment of wounds, first degree burns, bruises, and skin eruption. Several pharmacological studies have confirmed the healing of wounds treated with *Calendula officinalis* [18,19]. A previously published study regarding the effect of a 1% aqueous solution of ethanol extract obtained from marigold flowers on skin wounds of Wistar rats showed that wound epithelialization in the group treated with marigold extract has started on day 7 of treatment, and full recovery was observed within 14 days [17]. Our results with regard to both wound contraction and healing time are significantly better.

In addition, we observed the effect of stopping the process of infection within the lesion site when using *Calendula officinalis* ointment. Thus, histological improvement was observed starting with day three of treatment. In the untreated group, the presence of skin ulceration abscess and purulent exudates was detected, highlighting the existence of bacterial colonies and myositis versus the treated group, which is found to stop all these lesion events. In fact, a study on ethanol and methanol extracts of *Calendula officinalis* obtained from the flowers showed a good antibacterial effect on Gram-positive and Gram-negative strains but also an antifungal activity comparable to that of fluconazole [20]. 

In our study, from day 3 to day 6, we could observe that mature granulation tissue starts with a little scar fiber in the incision model, which demonstrates that marigold ointment stimulates an excessive production of collagen. This effect is beneficial when it comes to the collagenization in the area of the lesion, but the excessive production of collagen could lead to a fibrosis of the tissue, which is undesirable. In fact, the literature has shown that the gel based on *Calendula officinalis* can stimulate collagen deposits, helping to heal wounds [9].

Another study demonstrated the effect of two triterpenoid monoesters (faradiol myristate and faradiol palmitate) known for their anti-inflammatory effect [21]. Faradiol myristate (10 mg/mL) induced a lower fibroblasts proliferation than that obtained for extracts. It was concluded that not only triterpenes contribute to wound healing but also other compounds present in the *Calendula officinalis* extracts [22]. The biologically active compounds (flavonoids, carotenoids, terpenoids) possess anti-inflammatory effects by activating the transcription factor NF-kB. The studies reported that the lipophilic and the hydrophilic extracts prepared from marigold flowers accelerate the formation of the granulation tissue [23].

Our study has also demonstrated a high potential in terms of vessels neoformation. It is assumed that the wound healing potential of *Calendula officinalis* should be linked to the promotion of angiogenesis. *Calendula officinalis* has beneficial effects in angiogenesis through the induction of neovascularization and other proangiogenic factors (fibroblast growth factors - FGF, transforming growth factor beta - TGF-β) and angiogenic cytokines (interleukin-8 and tumor necrosis factor-α) [24].

The HPLC analysis led to the identification of chlorogenic acid as one of the major constituents in all three extracts. Recent studies revealed the importance of chlorogenic acid as a wound healing agent. Chen et al. demonstrated that the topical application of an ointment with 1% (*w/w*) chlorogenic acid can accelerate the process of excision wound healing similar to 1% (*w/w*) silver sulfadiazine ointment. This effect is explained by its capacity to increase collagen synthesis and its antioxidant effect [25].

Testing the effects of burdock plant extracts on skin lesions induced by incision, excision, and thermal burn is performed for the first time in this study. The literature quotes the effects of *Arctium lappa* (burdock) caffeoylquinic acids, which protects skin collagen from the effects of light radiation [26]. HPLC analysis showed that burdock extract contains a high amount of chlorogenic acid, supporting the literature reports.

Reducing the skin annexes found by us in this experiment is actually less important for this study, and it could become valuable information in the preparation of substances that stop hair growth on the body, but this requires further targeted studies on this topic.

In this study, we have obtained a skin collagenization, indicating a dermal tissue collagen action and lesion maturing. Given the lignans (arctigenin, arctiin) and polyphenols (caffeic and chlorogenic acids) contents, burdock leaves also exhibit antioxidant action [8].

For the group treated with yarrow ointment after 21 days of treatment, we have observed within the incision normal skin fragments with no damage. The excision model presented increased dermal collagenization. In the thermal burn model, 1/3 of the dermis had collagenous fibrosis and densification, and atrophic epidermis with rectilinear areas. *Achillea millefolium* (yarrow) has anti-inflammatory, hemostatic, antimicrobial, antiseptic, and antioxidant properties [27]. The results are consistent with studies already conducted, showing beneficial effects on burning lesions [28].

In addition to the advantages of bioactive compounds from herbal ointments, we also mention the use of various ointment bases, having a high content of lanolin. The base that incorporates the vegetal extracts has 50% lanolin (a product of natural origin) and 50% vaseline. This mix is preferred to the simple base, which has 10% lanolin and 90% vaseline. No parabens are used for conservation or other synthetic compounds. The natural active ingredients incorporated have proven healing properties, emollient, and re-epithelialization effect; they are biocompatible with the human body and have fewer side effects compared to synthetic compounds. Possible side effects could be sensitization reactions in patients who have allergies to members of the *Asteraceae* family, given that three of the four plants studied belong to this family [29]. The plants selected in the preparation of these ointments are widespread species, whose cultivation does not require special conditions and can ensure high amounts of plant material and thus can be easily adapted to industrial scale production. The promising results obtained in this experiment encourage testing the extracts on the diabetic wound model as well. 

Obtaining a high-quality product requires deep understanding and control of its rheological properties due to the strong relationship between flow and deformation characteristics of materials and their structure [30]. Thus, oscillatory and rotational rheological tests were used to evidence the macroscopic behavior of the complex systems and to correlate it with their structure. The rheological characterization of the studied ointments revealed important information about shear-induced structural modification affecting their texture, spreadability, sensory perception, and storage stability. The oscillatory tests allowed establishing and tailoring the right formulation for specific applications and good consumer acceptance by evidencing the correlation between the microstructure and the macroscopic behavior of the material.

For all the analyzed samples, the dynamic moduli are almost parallel for the whole frequency range with only an insignificant dependence on frequency. As mentioned in previous papers for different types of cosmetic products (creams, lotions), it can be supposed that for the studied ointments, the intermolecular interaction forces are forming a stable three-dimensional network. Moreover, the G′ values at low angular frequency (ω ≤ 0.1 rad/s) offer good indication about the so-called behavior “at rest” for the analyzed materials. With storage modulus values much higher than 10 Pa for all samples, more than satisfactory long-term stability of the ointments structures is expected [30,31]. 

## 4. Materials and Methods 

### 4.1. Plant Material

The plant material represented by Calendulae flos (*Calendula officinalis* L.), Millefolii herba (*Achillea millefolium* L.), Bardane folium (*Arctium lappa* L.), and Hippophae fructus (*Hippophae rhamnoides* L.) was collected from Balanesti (Gorj, Romania) and identified by specialists from the Department of Pharmaceutical Botany, “Grigore T. Popa” University of Medicine and Pharmacy, Iasi, Romania. Herbarium voucher samples (CO2016, AM2016, AL2016 and HR2016) were deposited in the Department of Pharmaceutical Botany. After collection, a part of the vegetal material was air-dried in a dark room at 23 ± 2 °C. 

### 4.2. Preparation of the Oily and Hydro-Alcoholic Extracts

The oily extracts of *Achillea millefolium*, *Arctium lappa,* and *Calendula officinalis* were prepared by macerating 50 g of fresh and ground plant material with 500 mL of virgin olive oil stored in brown bottles at room temperature for two weeks. The oily extract from *Hippophae rhamnoides* was obtained from a commercial source. The hydro-ethanolic extract of each plant was obtained by macerating 50 g of dried and ground plant in 500 mL ethanol 70% *v*/*v*, which were stored in dark bottles at room temperature for two weeks. After maceration, the extracts were filtered and kept in closed containers at 4 °C until testing.

### 4.3. Preparation of the Ointments

The ointment base was prepared by mixing 50 g of lanolin and 50 g of vaseline on a water bath (40 °C) to obtain a homogeneous base. The ointments have been obtained by adding 15 mL of hydro-alcoholic extract and 15 mL of oily extract of each plant in 100 g of ointment base, which was then homogenized in a water bath at 40 °C. 

### 4.4. Chemical Reagents

Gallic acid and sodium carbonate were purchased from Sigma-Aldrich (Steinheim, Germany). Folin–Ciocalteu’s phenol reagent was from Merck (Darmstadt, Germany), lanolin and vaseline were from Farma Chim (Ploiesti, Romania), and olive oil was from a commercial source.

### 4.5. Determination of Total Polyphenol Content

The content of total polyphenols from the alcoholic extracts was determined by the Folin–Ciocâlteu method, as previously described [32]. The total phenolic content of the extracts was expressed in gallic acid equivalents (mg gallic acid/g extract). All determinations were made in triplicate, and the results were expressed as the mean ± standard deviation.

### 4.6. HPLC Analysis

Polyphenolic compounds were quantified using an HPLC-UV-MS method, which was previously described [33]. Eighteen polyphenolic standards were used: caffeic acid, chlorogenic acid, p-coumaric acid, kaempferol, apigenin, rutin, quercetin, quercitrin, isoquercitrin, fisetin, hyperoside, myricetin (Sigma, Germany), ferulic acid, gentisic acid, sinapic acid, patuletin, luteolin (Roth, Germany), and caftaric acid (Dalton, Toronto, ON, Canada). Calibration curves in the 0.5–50 μg/mL range with good linearity (R^2^ > 0.999) were used to determine the concentration of polyphenols in plant samples. In order to separate caffeic and chlorogenic acids, a Zorbax SB-C18 reversed-phase analytical column (100 × 3.0 mm i.d., 3 µm particles) fitted with a guard column Zorbax SB-C18 was used. A mobile phase consisting of 0.1% acetic acid and acetonitrile was used. The identification of acids was made by MS/MS.

Methoxylated flavonoids were quantified through an LC-MS method described before [34]. Six standards were used: jaceosidin, eupatilin (ALB Technology, Hong Kong, China), casticin, acacetin, eupatorin, and hispidulin (Sigma, Germany). Calibration curves in the 0.02–6 μg/mL range with good linearity (R^2^ > 0.99) were used to determine the concentration of methoxylated flavones.

Phytosterols analysis was performed by a previously reported LC-MS method [34] using five standards: β-sitosterol, stigmasterol, campesterol, brassicasterol, and ergosterol, which were acquired from Sigma (Germany). Calibration curves of the sterols in the range of selected concentrations (0.06–6 μg/mL) showed a good linear correlation coefficient (R^2^ > 0.99).

### 4.7. Rheological Characterization of Ointments

Oscillatory and rotational rheological tests were carried out on a Physica MCR 501 (Anton Paar, Graz, Austria) modular rheometer equipped with a Peltier temperature control device. For all measurements, 50 mm diameter parallel plate geometry with serrated plates was used to avoid slippage of the sample. A thin layer of low viscosity silicone oil was applied on the edges of the sample to avoid moisture loss during studies. The rheology of the ointments was studied using both oscillatory (amplitude sweep, frequency sweep, time and temperature sweeps) and rotational shear measurements. All isothermal experiments were performed at two different temperatures (25 °C and 37 °C). Reproducibility was verified for all rheological tests on three samples of each analyzed ointment.

The rheological parameters used to characterize the ointments rheological behavior are the storage modulus G′ (measuring the elastic behavior of the material), loss modulus G″ (measuring the viscous behavior of the material), phase angle δ, damping or loss factor: tan δ = G″/G′ (revealing the ratio of the viscous and the elastic portion of the deformation behavior), and complex viscosity |η*| [30,35]. 

### 4.8. Animal Experiment

The research was carried out on Wistar adult male rats, weighing 220–250 g. The animals were housed in a temperature-controlled room, with light–dark cycles of 12:12 h; temperature (22 ± 0.5 °C) and relative humidity (65–70%) were kept constant. The experiment included 6 groups of Wistar rats (7-rat groups), namely: NC group—control group with incision, excision and thermal burn models, not treated; OB group—rats treated only with the ointment base; HR group—rats treated with *Hippophae rhamnoides* ointment, CO group—rats treated with *Calendula officinalis* ointment; AL group—rats treated with *Arctium lappa* ointment; AM group—rats treated with *Achillea millefolium* ointment. Each group was accommodated in a separate cage with free access to standard laboratory diet and water. Before proceeding with the experimental models, the animals were anesthetized with ketamine (100 mg/kg), and the hair on the dorsal part of the rats was shaved and cleaned with 70% alcohol.

Three experimental wound models (linear incision, circular excision, thermal burn) were performed on each animal. 

*The Incision Model.* Two paravertebral straight incisions (1 cm long) were made with a sterile surgical blade through the full thickness of the skin at a distance of 1.5 cm from the midline on each side of the vertebral column [36].

*The Excision Model.* The circular wound was made on the dorsal interscapular region of each animal by excising the skin with a 8 mm biopsy punch. The wounds were left open [36].

*The Thermal Burn Model.* The original tip of a soldering iron 40 W was replaced with a copper plate of 9 mm × 8 mm dimensions and brought to 100 °C. A high-performance non-contact thermometer laser (Raynger MX4, Richmond, IL, USA) was used to measure temperature. The device was placed vertically under its own weight on the back of the rats for 9 s in order to induce a deep-partial thickness burn. Immediately after each burn injury, the lesion was rinsed with normal saline solution [37]. 

Ointments and the ointment base were applied topically once a day for 21 days. The study assessed the clinical condition (edema, inflammatory infiltrate, congestion, crust formation and fall, the type of scar), determining the rate of wound contraction and re-epithelialization period. Histopathological examination was performed on days 1, 3, 6, 12, and 21 of treatment.

*Wound Healing Evaluation Parameters.* The calculation of the circular lesion area (excision wound) was determined by the formula A = πr^2^. The calculation of the elliptical area resulting from the lesion contraction process was calculated according to the formula: πa × b/4, where a represented a high axis and b represented a small axis. The wound contraction rate (WCR) was calculated as the percentage of the original wound size (50.27 mm^2^) for each animal on days 6 and 9, using the ratio (A_0_ − A_t_)/A_0_ × 100, where A_0_ is the initial wound area (day 0) and A_t_ is the wound area measured at day 6 and 9, respectively.

*Re-Epithelialization Period Measurement.* Wound closure was considered the endpoint of the epithelialization process and days needed to complete epithelialization were considered a period of epithelialization.

*Histological Examination.* Some skin fragments were removed in order to observe the epithelialization phase; for this process, animals were anesthetized intraperitoneally with ketamine (100 mg/kg). The collected samples were fixed in 10% buffered formalin for at least 24 h, dehydrated in solutions containing an increasing percentage of ethanol (60, 80, 90, and 98%, *v*/*v*), clarified with amylic alcohol, embedded in paraffin under vacuum, sectioned at 5 µm thickness, deparaffinized, and stained with hematoxylin–eosin (HE).

Scoring of the dermal inflammatory infiltrate was performed based on scores from the literature, adapted [38,39]. To establish the score, 5 microscopic fields with a magnification of 400 were examined, at the level of the papillary and reticular dermis, and chosen as representative for each case. The value of the final score was represented by the average value of the 5 fields analyzed. A score of 0 represented no inflammatory infiltrate, a score of 1 represented mild/rare/occasional inflammatory infiltrate (<10 lymphocytes/high-power field (HPF), a score of 2 represented moderate/focal inflammatory infiltrate (11–30 lymphocytes/HPF), and a score of 3 represented severe inflammatory infiltrate (numerous lymphocytes > 30/HPF).

All experimental procedures performed on laboratory animals (Wistar rats) were respected in accordance with animals’ bioethics guidelines and with European Council Directive 2010/63/EU. The research was approved by the Ethics Committee of “Grigore T. Popa” University of Medicine and Pharmacy (registration no. 6349/2015).

### 4.9. Statistical Analysis

The data obtained from the excision wound model were analyzed by one-way ANOVA followed by a Bonferroni post test. Statistical analysis was performed using SPSS version 15.0, where *p* < 0.05 was considered statistically significant.

## 5. Conclusions

In conclusion, the market success of a cosmetic or pharmaceutical product strongly depends on its formulation and the rheological behavior can emphasize its suitability for a specific destination. The correct formulation and choice of ingredients allow the product to flow easily from the container (yield point, τ_0_), to prevent sedimentation of solid particles during storage, and to determine a good stability (zero shear viscosity, η_0_) and applicability on skin (pseudoplastic behavior). The rheological behavior of materials allows estimation of the sensory properties of an ointment, cream, or lotion. Furthermore, the uniformity and integrity of the film developed on the skin can be estimated to favor important applications in medicine and controlled drug release. 

Phytotherapeutic ointments based on *Hippophae fructus, Calendulae flos, Bardanae folium,* and *Millefolii herba* exerted a wound healing and re-epithelization effect on incision, excision, and thermal burn wounds. The skin lesions with loss of substance evolved in a mature, supple, foldable scar, in comparison with the untreated group that presented a rigid, hypertrophic scar. In addition, the vascularization, through the process of angiogenesis highlighted by the anatomical–pathological examination, was resumed. The final functional and aesthetical result was satisfactory. The most effective ointment proved to be the one based on *Arctium lappa*, followed by that of *Calendula officinalis*, both in terms of area of injury and in terms of wound contraction rate. All herbal ointments showed histologically efficient dermal collagenization with the appearance of normal granulation tissue after 21 days of topical treatment.

## Figures and Tables

**Table 1:** Concentration of phenolic compounds (µg/mL) in the extracts.

Polyphenols	*Arctium lappa*		*Achillea millefolium*		*Calendula officinalis*	
	MS	Conc.	MS	Conc.	MS	Conc.
Gentisic acid	*					
Caffeic acid		0.74		1.63		1.74
Chlorogenic acid	*	339.06	*	238.57	*	46.35
p-coumaric acid					*	-
Ferulic acid	*	0.153				
Hyperoside			*	-		
Isoquercitrin			*	-	*	9.905
Rutoside	*	99.279	*	1.599	*	7.983
Quercitrin			*	1.487	*	-
Quercetin			*	0.394		
Luteolin			*	7.796		
Apigenin			*	3.226		

* MS—MS qualitative determination; UV signal < LoQ (limit of quantification) does not allow the quantitative determination of these substances. Not found: kaempferol, fisetin, myricetin, patuletin, sinapic acid, and caftaric acid.

**Table 2:** Concentration of methoxylated flavonoids (ng/mL) in the extracts.

Methoxylated Flavonoids	*Arctium lappa*	*Achillea millefolium*	*Calendula officinalis*
Acacetin	-	107.12	-
Casticin	-	63.56	-
Hispidulin	-	877.78	-

Not found: eupatorin, eupatilin, and jaceosidin.

**Table 3:** Concentration of sterols (µg/mL) in the extracts.

Sterols	*Arctium lappa*	*Achillea millefolium*	*Calendula officinalis*
Stigmasterol	-	1.036	1.584
Beta-sitosterol	1.139	7.700	3.264
Campesterol	-	0.3121	0.116
Brassicasterol	-	0.166	-

Not found: ergosterol.

**Table 4:** Evaluation of wound area, re-epithelialization area, and wound contraction rate for the excision model.

Experimental Groups	Wound Area (mm^2^)			The Re-Epithelialization Area (mm)	WCR (%) (Mean ± SEM)	
	Day 0	Day 6	Day 9	Day 12	Day 6	Day 9
HR Group	64	36	7.5	2 × 2.5	69.38 ± 0.83	88.23 ± 0.49
	(8 × 8)	(6 × 6)	(2.5 × 3)			
CO Group	64	28	5	1.5 × 2	57.29 ± 1.80 *	92.70 ± 0.47 *
	(8 × 8)	(4 × 7)	(2 × 2.5)			
AL Group	64	15	3.4	1 ×2	77.66 ± 0.68	94.29 ± 0.26
	(8 × 8)	(3 × 5)	(1.7 × 2)			
AM Group	64	32.5	7.5	2 × 3	51.30 ± 1.14	87.50 ± 0.41
	(8 × 8)	(5 × 6.5)	(2.5 × 3)			
OB Group	64	45.5	40.8	-	26.74 ± 2.13 *	33.79 ± 2.21 *
	(8 × 8)	(6.5 × 7)	(6 × 6.8)			
NC Group	64	61.6	56.25	-	5.78 ± 1.86	14.85 ± 2.95
	(8 × 8)	(7.7 × 8)	(7.5 × 7.5)			

* *p* < 0.001.

**Table 5:** Histological evaluation of the tissue samples at day 3.

Experimental Groups	Experimental Models		
	Incision	Excision	Thermal Burn
NC Group	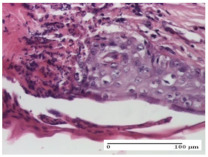	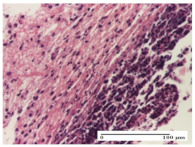	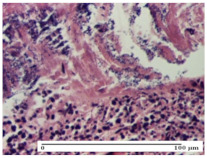
	abscess with epidermal ulceration, Score 3	purulent exudate, Score 3	ulceration and bacterial colonies, Score 3
OB Group	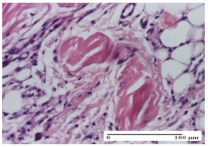	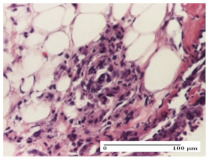	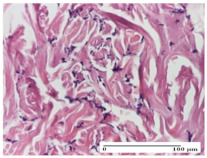
	inflammatory infiltration in hypodermis, striated muscle, Score 1	adipose tissue with inflammation, Score 2	dermal edema, Score 1
HR Group	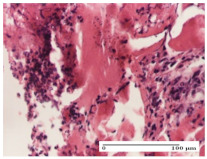	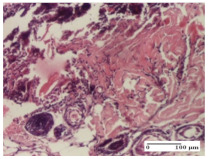	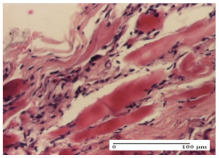
	lymphocytes arranged around capillary and intramuscular blood vessels, Score 3	numerous lymphocytes, polymorphonuclear cells, Score 3	inflammatory infiltrate in the muscular layer, Score 1
CO Group	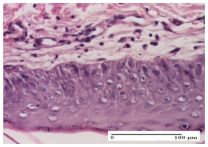	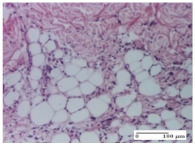	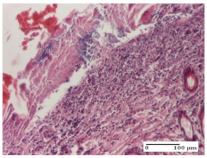
	lymphocytes in superficial dermis, Score 1	fibrosis and lymphocytic infiltration, Score 1	ulcerated epithelium, Score 3
AL Group	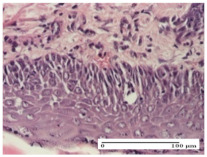	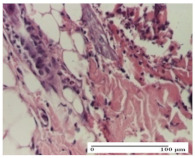	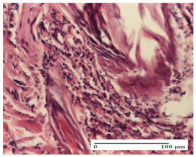
	vascular congestion and perivascular lymphocytes, Score 1	inflammatory infiltrate in the hypodermis, Score 1	periadnexal inflammatory infiltrate, Score 2
AM Group	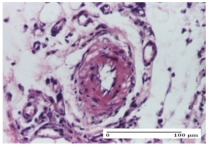	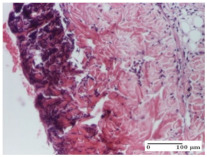	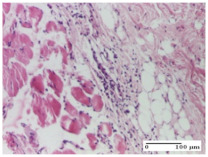
	perivascular inflammatory infiltrate, Score 1	ulcerated epidermis, Score 3	lymphocytes, hypodermis and muscle, Score 1

Inflammatory infiltration scoring: Score 0 (no inflammatory infiltrate), Score 1 (mild inflammatory infiltrate), Score 2 (moderate inflammatory infiltrate), Score 3 (severe inflammatory infiltrate).

**Table 6:** Histological evaluation of the tissue samples at day 6.

Experimental Group	Experimental Models		
	Incision	Excision	Thermal Burn
NC group	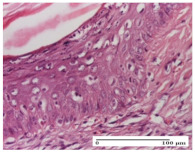	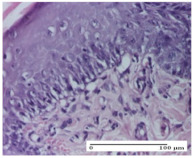	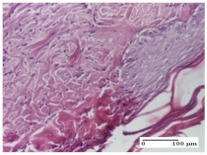
	vacuolar degeneration, epidermal acanthosis, Score 1	spongiosis, edema, lymphocytes, fibroblasts, Score 1	vacuolar degeneration in the epidermis, epidermal ulceration, Score 1
OB group	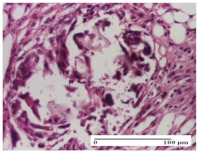	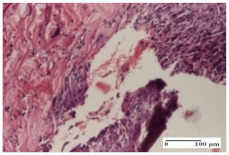	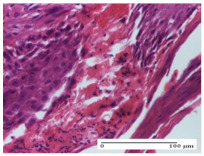
	multinucleated foreign-body giant cells, Score 2	epidermal ulceration, Score 3	hemorrhage and abscess, Score 2
HR group	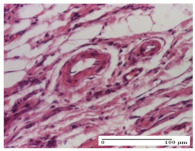	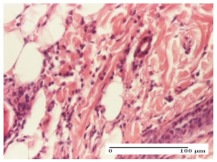	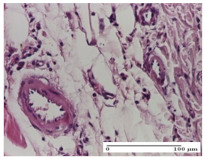
	perivascular lymphocytes, Score 1	lymphocytes, Score 1	perivascular lymphocytes, Score 1
CO group	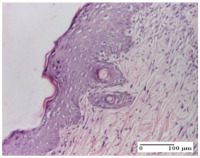	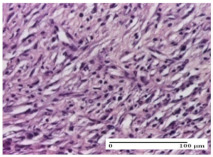	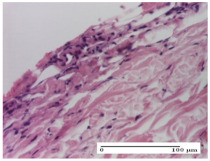
	superficial dermal collagenization, Score 2	area undergoing connective tissue organization, Score 2	lymphocytes in the dermis, Score 1
AL group	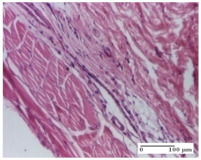	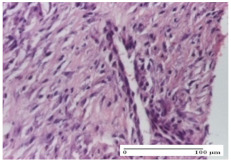	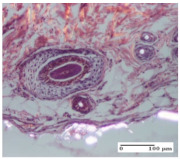
	normal striated muscle, Score 1	granulation tissue in course of conjunctive organization, Score 1	normal hair, detail in polarized light microscopy, Score 0
AM group	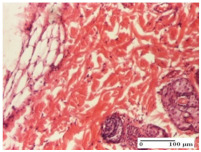	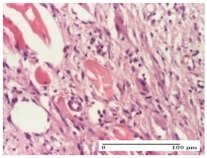	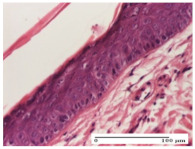
	rare lymphocytes, Score 0	residual myocytes and inflammatory infiltrate, Score 2	rare lymphocytes, Score 1

Inflammatory infiltration scoring: Score 0 (no inflammatory infiltrate), Score 1 (mild inflammatory infiltrate), Score 2 (moderate inflammatory infiltrate), Score 3 (severe inflammatory infiltrate).

**Table 7:** Histological evaluation of the tissue samples at day 12.

Experimental Groups	Experimental Models		
	Incision	Excision	Thermal Burn
NC group	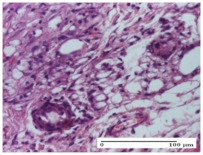	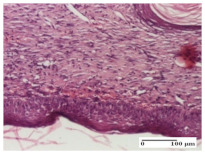	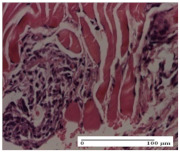
	lymphocytic infiltrate in the hypodermis, Score 2	congestion, lymphocytic infiltrate in the dermis, Score 2	inflammatory infiltrate, striated muscle, Score 2
OB group	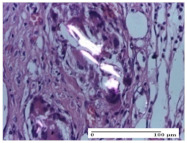	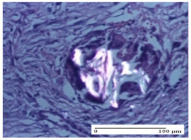	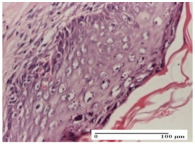
	foreign body-type multinucleated giant cell, detail in polarized light microscopy, Score 1	multinucleated giant cell, detail in polarized light microscopy, Score 1	acanthosis and vacuolar degeneration, Score 1
HR group	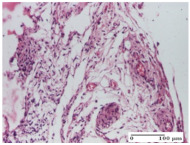	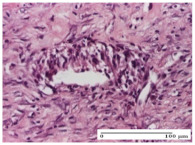	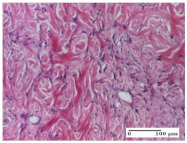
	lymphocytes, capillary congestion, Score 2	lymphocytes, fibroblasts, Score 2	dermal collagenization, Score 1
CO group	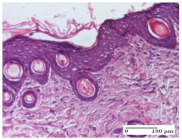	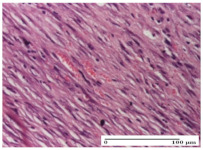	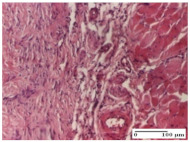
	mild dermal collagenization (collagen fibers with a horizontal and homogenized arrangement), Score 1	collagenization, Score 1	significant dermal collagenization, Score 1
AL group	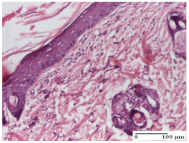	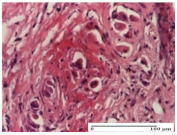	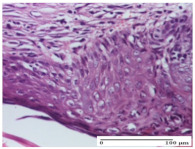
	rectilinear epidermis; lymphocytes in the superficial dermis, Score 1	residual myocytes, in the organized conjunctival area detail, Score 1	lymphocytes in the superficial dermis, Score 1
AM group	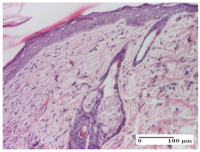	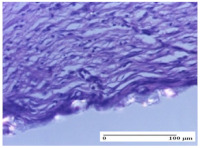	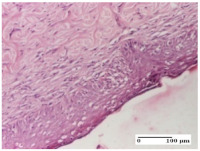
	mild dermal collagenization, Score 1	significant dermal collagenization, detail in polarized light microscopy, Score 1	dermal collagenization, Score 1

Inflammatory infiltration scoring: Score 0 (no inflammatory infiltrate), Score 1 (mild inflammatory infiltrate), Score 2 (moderate inflammatory infiltrate), Score 3 (severe inflammatory infiltrate).

**Table 8:** Histological evaluation of the tissue samples at the end of treatment (day 21).

Experimental Groups	Experimental Models		
	Incision	Excision	Thermal Burn
NC group	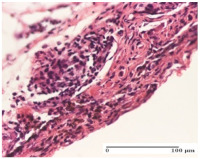	** 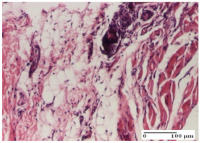 **	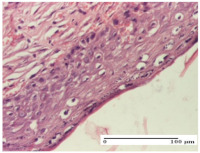
	rich inflammatory infiltrate, predominantly lympho-plasmacytic, Score 3	dermal edema, Score 2	vacuolar degeneration, Score 1
OB group	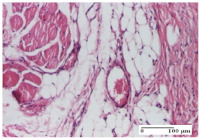	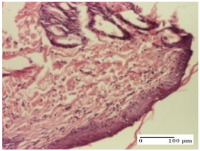	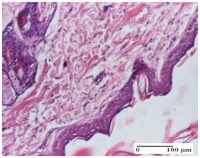
	vascular congestion, Score 1	mild edema, Score 1	lymphocytes, Score 1
HR group	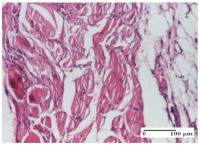	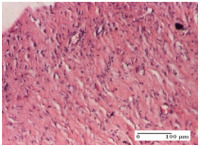	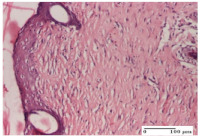
	muscle, Score 1	dermal collagenization, Score 1	dermal collagenization, Score 1
CO group	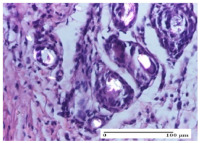	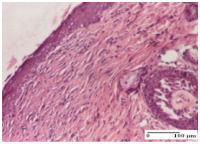	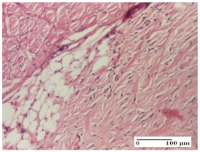
	moderate lymphocytic infiltrate; medium dermal collagenization, Score 1	dermal collagenization, Score 1	significant dermal collagenization, Score 1
AL group	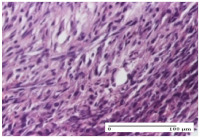	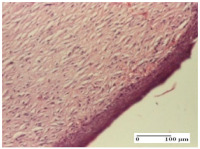	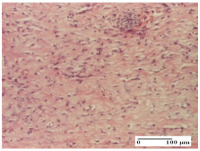
	mature granulation tissue, Score 2	mature granulation tissue, Score 1	mature granulation tissue, Score 1
AM group	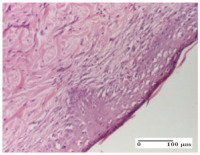	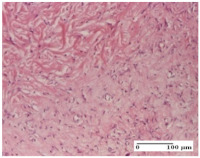	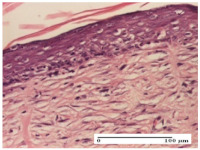
	dermal collagenization, Score 1	dermal collagenization, Score 1	dermal collagenization, Score 1

Inflammatory infiltration scoring: Score 0 (no inflammatory infiltrate), Score 1 (mild inflammatory infiltrate), Score 2 (moderate inflammatory infiltrate), Score 3 (severe inflammatory infiltrate).
